# Endometrial Cancer in Women with Adenomyosis: An Underestimated Risk?

**DOI:** 10.22074/ijfs.2020.44413

**Published:** 2020-10-12

**Authors:** Antonio Simone Laganà, Marco Scioscia

**Affiliations:** 1. Department of Obstetrics and Gynecology, "Filippo Del Ponte" Hospital, University of Insubria, Varese, Italy; 2. Department of Obstetrics and Gynecology, Policlinico Hospital, Abano Terme, Padua, Italy

Endometrial carcinoma (EC) has a worldwide incidence of 8.4 per 100,000 persons per year and is a leading cause of cancer mortality in women in developed countries after ovarian and cervical cancer (1). Among the gynecological diseases, polycystic ovary syndrome and uterine leiomyomas have been associated with a potential increased risk to develop EC, whereas less robust data are available for endometriosis and adenomyosis (2). The association between EC and adenomyosis, defined by the presence of ectopic endometrial glands and stroma within the myometrium, is still debated: on the one hand, adenomyosis is found with an incidence of 10 to 18% in EC specimen at final histology after hysterectomy (3). On the other hand, accumulating evidence suggests that these two diseases share several altered molecular pathways, which lead to increased angiogenesis, abnormal tissue growth, and invasion. In details, adenomyosis and EC are both associated with a local microenvironment characterized by high level of vascular endothelial growth factor, platelet-derived growth factor, increased production of reactive species of oxygen and pro-inflammatory cytokines, KRAS mutations and, to a lesser extent, progesterone-resistance, epithelialmesenchymal transition and fibroblast-to-myofibroblast trans-differentiation (4). All these elements can cause growth and reduced apoptosis rate of endometrial stromal cells. Interestingly, accumulating evidence suggests that EC may arise due to incessant proliferative stress of endometrial stromal cells at the junctional zone endometrium (5), the same anatomical and histological location hypothesized to give origin to adenomyotic foci from oligoclonal stromal cells. In this scenario, recently a large epidemiological dataset compared endometrioid EC co-existing with adenomyosis and endometrioid EC arising from adenomyosis with EC without adenomyosis (6). According to this analysis, EC arising from adenomyosis was associated with significantly younger onset ages and better survival than other cases where adenomyosis was just co-existing. This distinctive behavior between the two conditions may suggest that when EC arises from adenomyotic microenvironment, the degenerated stromal cells could have a less aggressive phenotype and could more susceptible to hormonal influence. Although speculative, the differences between EC arising from, or just co-existing with, adenomyosis may be a key element to understand also the potential different responses to hormonal drugs such as medroxyprogesterone and levonorgestrel-release intra-uterine devices, especially in the scenario of fertility-sparing approach in very selected patients with early-stage disease. A correlation between EC and obesity with metabolic syndrome (i.e. polycystic ovary syndrome) was proven (1), so further studies are required to evaluate a correlation between adenomyosis and these factors. On that basis, we solicit both future large epidemiological studies and molecular investigations to clarify whether with EC arising from adenomyosis or just co-existing with it may need different therapeutic strategies.

## References

[1] Siegel RL, Miller KD, Jemal A (2019). Cancer statistics, 2019. CA Cancer J Clin.

[2] Yuan H, Zhang S (2019). Malignant transformation of adenomyosis: literature review and meta-analysis. Arch Gynecol Obstet.

[3] Vercellini P, Viganò P, Somigliana E, Daguati R, Abbiati A, Fedele L (2006). Adenomyosis: epidemiological factors. Best Pract Res Clin Obstet Gynaecol.

[4] Inoue S, Hirota Y, Ueno T, Fukui Y, Yoshida E, Hayashiet T (2019). Uterine adenomyosis is an oligoclonal disorder associated with KRAS mutations. Nat Commun.

[5] Tanos V, Lingwood L, Balami S (2020). Junctional zone endometrium morphological characteristics and functionality: review of the literature. Gynecol Obstet Invest.

[6] Chao X, Wu M, Ma S, Tan X, Zhong S, Bi Y (2020). The clinicopathological characteristics and survival outcomes of endometrial carcinoma coexisting with or arising in adenomyosis: a pilot study. Sci Rep.

